# Cancer Incidence among Healthcare Workers in Cancer Centers: A 14-Year Retrospective Cohort Study in Thailand

**DOI:** 10.29024/aogh.2324

**Published:** 2018-08-31

**Authors:** Chatchai Ekpanyaskul, Suleeporn Sangrajrang

**Affiliations:** 1 Department of Preventive and Social Medicine, Faculty of Medicine, Srinakharinwirot University, TH; 2 Research unit, National Cancer Institute, Department of Medical Services, Ministry of Public Health, TH

## Abstract

**Objective:**

To identify the situation and possible work-related cancer risks among healthcare workers in cancer centers.

**Methods:**

This research was a 14-year retrospective cohort study of 2,331 healthcare workers at the National Cancer Institute and 7 regional cancer centers in Thailand. The study period consisted of a total of 18,939 person-years of observation. The demographic data, such as occupation and work area were collected by self-administered questionnaires or by use of a proxy. The cases were identified by the diagnoses of physicians. The incidence rates for each type of cancer, occupation and work area among the population of this study were compared with the general working population, based on national cancer statistics. The results were reported in terms of Standard Incidence Ratio (SIR) and a 95% confidence interval (CI), using Fisher’s exact method.

**Findings:**

There were 12 different types of cancer identified in 35 cases during the 14 years of the study and breast cancer was found to be at the highest number. The overall cancer incidence rates were 221.04 and 173.43 per 100,000 person-years, in males and females, respectively. Leukemia showed statistically significant levels of high SIR among the female healthcare staffs (SIR = 11.54; 95% CI = 2.38–33.72). With regard to occupation, only the male physicians showed significant SIR = 6.02; 95% CI = 1.41–19.93, while this study did not identify significant SIR levels in any of the work areas.

**Conclusions:**

This study found that the risk of leukemia was higher than expected among healthcare workers and that physicians may have an increased risk of cancer compared to the general working population, which may be a work-related reflex. However, interpretations should be made with caution due to the small number of cases.

## Background

The working environment is an important factor that influences the health of workers, particularly in a hospital setting. Hospitals are a unique kind of workplace, with a multitude of working processes and complexities. They usually encompasses a broad spectrum of occupations and exposes workers to a variety of health hazards or health risks. Bernardino Ramazzini described and commented on this phenomenon in “The Diseases of Workers,” a medical text from 1700. Even at this time, healthcare work was recognized as one of the oldest professions, with an at-risk population and a hazardous work environment [1]. For this population group, occupational health is an important aspect of societal reintegration. Previous reports indicated that healthcare workers (HCWs) suffered from the second highest rates of injury and illness and that these figures continue to rise. Every year, there are also economic losses due to occupational injuries or diseases [2]. Depending on the specific work environment, HCWs are at risk from exposure to classical hazards, such as physical, chemical and biological hazards, as well as more modern hazards, including shift work, stress or violence experienced during patient care.

Work-related cancer is one of the emerging occupational health problems of this decade, continuing to rise and resulting in numerous social concerns. Many HCWs have been exposed to a range of specialty-specific occupational carcinogens in the course of their daily duties. The situations which raised the most concern were physicians exposed to blood-borne carcinogens during certain procedures [3], technicians exposed to radiation from diagnostic and therapeutic procedures [4], sterile workers being exposed to ethylene oxide [5], oncology nurses and pharmacists involved in the preparation and administration of antineoplastic agents [6], clinical technicians utilizing formaldehyde or carcinogenic chemicals for laboratory activities [7] and exposure to very dangerous secretions for the housekeeping staffs [8]. Based on these examples, all of these hospital hazards are IARC-classified as being carcinogenic for humans (Class I) [9,10,11]. While previous studies have demonstrated that many occupations in an industrial setting result in exposure to carcinogens that may be associated with various types of cancer. However, the research regarding this outcome among HCWs was limited.

In Thailand, HCWs are a large occupational sector of the working population. The data from the Ministry of Public Health in Thailand revealed that there were more than 200,000 HCWs working in a hospital setting. The job categories included clinical care, nursing care, clinical support, facility support and the back office [12]. Of these, the majority of the occupations included physicians, nurses and work that required multitasking and various potential exposures to carcinogens. Over the past two decades there have been an increasing number of cancers of an unknown etiology among HCWs, such as the increasing rate of liver cancer among physicians or the high rate of breast cancer among nurses. There has been social debate on whether or not the rate of cancer among HCWs are due to the previously mentioned carcinogens, which may occur in the environment of a working hospital. However, to date these issues have never been thoroughly explored. The high number of cases may be an epidemic of cancers or occurring due to a lack of expedient access to care.

The objective of this study was to describe the real-life cancer cases experienced by Thai HCWs by describing the epidemiology and comparing the population of the study to the general working population. Also, the study sought to identify which occupation and work environment were possibly related to cancer in this population. The results could be an aid to understanding the health problems of HCWs. This is crucial to the development of a strategic occupational health plan for cancer prevention in this occupation.

## Methods

This study was conceived in line with the Declaration of Helsinki. After the study protocol was approved by the Ethics Committee of Thai National Cancer Institute, the study was conducted by a retrospective cohort study in the Thai National Cancer Institute and seven regional cancer centers. The regional cancer centers consisted of one regional center in the north, two regional centers in the northeast, two regional centers in the central region, one regional center in the east and one regional center in the south of Thailand. The data retrospective was conducted from 1995 to 2008. The cohort included HCWs who worked at the National Cancer Institute since 1995 or in open-service at each regional center. There was a cohort of 2,572 HCWs who worked during this period. From this population, only 2,331 people were eligible to be included in this study. HCWs with less than a single year of employment at the National Cancer Institute and the seven regional cancer centers, in all regions of Thailand were excluded (90.6% coverage rate). The study period contributed a total of 18,939 person-years of observation.

The person-years at risk in the cohort study was calculated from each HCW from 1995 or from their first year of employment to the end of 2008, their retirement date or the date of their cancer diagnosis. The demographic data, such as age, gender, area of residence and occupational history, such as the time-period since starting work, occupation and work area were collected by self-administered questionnaires or by the use of proxies that were rechecked with institutional records and available data on the staff. The cases were identified by the medical histories and diagnoses from the time of treatment. The type of cancer was a primary cancer, rather than a metastasis. The latency period was calculated from the date started work in the National Cancer Institute or regional cancer centers until a cancer diagnosis. With regard to these periods, if the latency period was greater than five-years for hematological cancer or greater than ten-years for solid organ cancer, a work-related cause was suspected. The epidemiology of cancer cases were described using descriptive statistics, including frequency and percentage.

The incidence rates of overall cancer in the study population were reported by incidence density rate, which was expressed by the number of cancer cases per 100,000 person-years. Factors such as gender, area of residence and the types of cancer were compared to data on the general Thai working population, from 20 to 59 years of age and with indirect adjustment standardization. With regard to the small observed number and Poisson distribution, the Standard Incidence Ratio (SIR) and a 95% confidence interval (CI) for SIR was calculated and reported by the use of an Open Source Epidemiologic Statistics for Public Health (OpenEpi) online analysis calculator and Fisher’s exact method [13].

The SIR is defined as the ratio of the actual observed number (O) of each variable among HCWs divided by the expected number (E) of that variable. The expected number were found in the national statistical data from Cancer in Thailand Volume VII, 2007–2009 [14]. Due to the small number of cases for occupation and work-site analysis, this study combined all of the different types of cancer into a single category prior to SIR analysis. For the purposes of interpretation, both the SIR and the 95% confidence interval (CI) > 1 were calculated and it indicated that the occurrence of particular cancers and the fact that the previously mentioned variables among HCWs was higher than that of the general working population and had a statistical significance.

## Findings

Over the past 14 years, there were notified cancer cases among a total of 35 HCWs, who developed 12 different types of cancer. The overall cancer incidence density rates were 221.04 and 173.43 per 100,000 person-years, for males and females, respectively. In total, the 35 cases identified during the study period included ten different organs and are as follows in order of the highest to lowest: breast (9 cases), hematological system (6 cases), colorectal system (5 cases), lungs (5 cases), liver (3 cases), cervix (2 cases), thyroid (2 cases), ovaries (1 case), kidneys (1 case), and brain (1 case). With regard to the HCWs studied in this research, 63% of the patients are still alive and 60% survived the five-year period of data collection. The average age at diagnosis was 46.1 ± 9.2 years.

There were 25 females and 10 males with a female-to-male ratio of 2.5:1. The incidence of cancer in Bangkok and the regional area was shown in Table 1. The major occupation of cases was among the nurses (28.6%). Approximately 65.7% of cases (23 out of 35) were identified as working in clinical service areas. The year of diagnosis of these cases was spread over the study period. However, these cases occurred more frequently over the past decade than previously. The typical age of a patient at the time of diagnosis for most cases in this study was similar to that of the general population. The distribution of the latency period—the number of years from beginning work to diagnosis—ranged from 2 to 35 years. It was found that 26 out of 35 cases (74.3%) had a latency ≥5 years for hematological cancer or ≥10 years for solid organ cancer.

**Table 1:** The incidence rate, the Standard Incidence Ratio (SIR) and its 95% confidence interval (CI) stratified by gender and area of residence.

Gender/Area of residence	Incidence rate per 100,000 person-years	Person-years	O	E	SIR	95%CI

**Male**	221.04	4524	10	6.27	1.60	0.77–2.93
–Bangkok	404.20	1237	5	1.66	3.01	0.98–7.03
–Regional area	152.11	3287	5	4.20	1.19	0.39–2.78
**Female**	173.43	14415	25	24.71	1.01	0.65–1.49
–Bangkok	175.60	5683	10	12.13	0.82	0.40–1.52
–Regional area	171.78	8732	15	15.42	0.97	0.54–1.60

When compared with the general population, the incidence rate of overall cancer among male and female healthcare workers and the stratification of area by residence was not statistically significantly higher than that of the general working population. (Table 1) For each type of cancer, the SIR of hematological cancer-leukemia was the only single type of cancer that was statistically significantly higher than the general population. With regard to the subgroup analysis, only suspected work-related cases used the latency period ≥5 years for hematological cancer and ≥10 years for solid organ cancer, while leukemia still remained statistically significant (Table 2). An analysis of occupation and working area risk revealed that only male physicians had statistically significant and higher levels of SIR when compared to the general working population. The details are as follows in Tables 3 and 4.

**Table 2:** The Standard Incidence Ratio (SIR) and its 95% confidence interval (CI) of each type of cancer and suspected work-related cancer among HCWs.

Type of cancer	Total cases				Selected only case who had latency period ≥5 years for hematological cancer or ≥10 years for solid organ cancer			

	O	E	SIR	95%CI	O	E	SIR	95%CI

**Male (n = 10)**								
–colon	2	0.34	6.06	0.73–21.89	1	0.34	2.94	0.07–16.39
–rectum	1	0.23	4.55	0.12–25.33	1	0.23	4.55	0.12–25.33
–liver	2	1.93	1.04	0.13–3.74	2	1.93	1.04	0.13–3.74
–lung	3	0.85	3.53	0.73–10.31	3	0.85	3.53	0.73–10.31
–lymphoma	2	0.27	7.41	0.90–26.76	2	0.27	7.41	0.90–26.76
**Female (n = 25)**								
–colon	2	1.01	1.97	0.24–7.13	2	1.01	1.97	0.24–7.13
–liver	1	2.11	0.47	0.01–2.64	1	2.11	0.47	0.01–2.64
–lung	2	1.48	1.35	0.16–4.88	0	1.48	<0.01	<0.001–2.49
–breast	9	6.53	1.38	0.63–2.62	6	6.53	0.92	0.34–2.00
–cervix	2	3.93	0.51	0.06–1.84	2	3.93	0.51	0.06–1.84
–ovary	1	1.38	0.72	0.02–4.03	0	1.38	<0.01	<0.001–2.67
–kidney	1	0.10	10.31	0.26–57.44	1	0.10	10.31	0.26–57.44
–brain	1	0.39	2.57	0.07–14.32	0	0.39	<0.01	<0.001–9.46
–thyroid	2	1.01	1.98	0.24–7.15	1	1.01	0.99	0.03–5.54
–lymphoma	1	0.69	1.45	0.04–8.08	1	0.69	1.45	0.04–8.08
–leukemia	3	0.26	11.54	2.38–33.72	3	0.26	11.54	2.38–33.72

**Table 3:** The Standardized Incidence Ratio (SIR) and its 95% confidence interval (CI) in different healthcare worker occupational groups, stratified by gender.

Occupation	Male				Female			

	O	E	SIR	95%CI	O	E	SIR	95%CI

–administrator/clerk	1	1.01	0.99	0.03–5.52	8	5.94	1.35	0.58–2.65
–physician	3	0.44	6.02	1.41–19.93	1	0.47	2.13	0.05–11.85
–nurse/assistant nurse	0	0.49	<0.01	<0.001–7.53	10	9.36	1.07	0.51–1.96
–radiological technician	1	0.70	1.43	0.04–7.96	2	0.73	2.74	0.33–9.90
–laboratory technician/researcher	1	0.50	2.00	0.05–11.14	0	1.59	<0.01	<0.001–2.32
–pharmacist	1	0.29	3.45	0.09–19.21	0	0.52	<0.01	<0.001–7.09
–maintenance technician	2	0.89	2.25	0.27–8.12	–	–	–	–
–supportive care such as assistant patient worker, housekeeper	1	1.35	0.74	0.02–4.13	3	5.32	0.56	0.12–1.65
–others i.e. nutritionist, driver, health educator	0	0.61	<0.01	<0.001–6.05	1	0.76	1.32	0.03–7.33

“–” = no subject in this category.

**Table 4:** The Standardized Incidence Ratio (SIR) and the 95% confidence interval (CI) among healthcare staff and working areas stratified by gender.

Working area	Male				Female			

	O	E	SIR	95%CI	O	E	SIR	95%CI

–office	1	1.49	0.67	0.02–3.74	8	7.37	1.09	0.47–2.14
–out-patient department	2	0.55	3.64	0.44–13.14	5	3.33	1.50	0.49–3.50
–in-patient department/intensive care unit	0	0.44	<0.01	<0.001–8.38	5	6.75	0.74	0.24–1.73
–operating room	0	0.17	<0.01	<0.001–21.70	1	0.66	1.52	0.04–8.44
–radiation department	2	1.07	1.87	0.23–6.75	4	2.34	1.71	0.47–4.38
–maintenance unit	2	0.90	2.22	0.27–8.03	–	–	–	–
–laboratory/research unit	1	0.64	1.56	0.04–8.71	0	1.84	<0.01	<0.001–2.00
–chemotherapeutic unit	1	0.10	10.0	0.25–55.72	1	0.54	1.85	0.05–10.32
–pharmacy unit	1	0.30	3.33	0.08–18.57	0	0.71	<0.01	<0.001–5.20
–supply/laundry unit	0	0.24	<0.01	<0.001–15.37	0	0.22	<0.01	<0.001–16.04
–others i.e. transport unit, security, catering	0	0.35	<0.01	<0.001–10.54	1	0.95	1.05	0.03–5.87

“–” = no subject in this category.

## Discussion

The improvement of the public health system may reduce many causes of early death. DALY has increased globally for long latency diseases such as cancer [15]. There were several risk factors related to cancer, such as the environmental aspects and the genetic factors. Of these factors, occupation is the one risk factor that has become an increasing trend [16]. It can be easily prevented, if the exact type of carcinogen exposure can be correctly identified [17]. There is increasing concern among Thai HCWs about this problem due to the presence of multiple carcinogens in the healthcare setting. This is one of the few studies to consider the risk of cancer among HCWs.

The results of this longitudinal study in the tertiary healthcare setting in Thailand demonstrated that the incidence density rate of overall cancers among both males and females, which were higher than the national statistics but had no more significance as the category of residence. Although the majority of the healthcare workforce was female, and the overall incidence density rate of male cancers occurred at a higher rate than their female counterparts in other workforces.

When examining each type of cancer, this study found that the incidence density rate of leukemia among female HCWs was the only form of cancer with remarkable statistical significance. This type of cancer had a low incidence in general among the Thai population, but occurred at a high rate among HCWs [14]. The observed cancer risk-taking behavior gave reason to hypothesize a possible occupational influence. This was supported by other studies and HCWs in some occupations which had hematologic carcinogen exposure, genotoxicity in their white blood cells and were at risk for leukemia [18,19,20]. Moreover, all of the leukemia cases had a latency period of greater than five-years which may be compatible with and support possibly work-related causes. However, the factor of specific occupational exposure may require further exploration in the future.

The incidence of liver cancer was of concern due to a previous policy against Hepatitis B immunization among HCWs [21], while female breast cancer was recently related to shift work [22,23,24]. The high number of cases was due to the large proportion of female HCWs in this study. Although their level of SIR increased, it was not deemed to be statistically significant.

Of all of the cancers combined in this study, only male physicians had significantly high incidence density rate of cancer compared to the general Thai working population. These results were also consistent with other Western and Eastern studies [25,26,27]. Over the past two decades, most developing countries, such as Thailand, suffered from a lack of physicians and had high expectations of the medical attention provided by society. In other words, they have heavy workloads, come into close contact with infectious patients, are on duty at night and experience more stress, have short and irregular sleeping patterns, they fail to exercise and indulge in negative health behavior risks. This is particularly true of the males, with behaviors such as smoking, drinking alcohol and imbalanced food consumption, which may lead to obesity. Some behavioral risks occurred and had a cumulative effect on them, since they were medical students [28,29]. Moreover, in these periods, there were no occupational health system for HCWs, such as vaccinations, periodic risk-based examinations and proper personal protective equipment. All of these problems could increase susceptibility or vulnerability to cancer among male physicians. While in the work environment, it was found that the chemotherapeutic unit had the highest SIR among both males and females, in comparison to other working areas. Currently, there are realized chemotherapeutic drugs as a work hazard and the IARC has classified many chemotherapeutic drugs as a carcinogen [9]. However, this study did not find a significantly higher incidence density rate of cancer than the general working population in other working areas in hospital. However, preventive measures in high risk working areas and surveillance of this population must be conducted before this problem becomes an epidemic.

This study may be significant in improving occupational health services for work-related cancer prevention. However, there are some limitations which should be taken into consideration before any attempts at interpretation. Firstly, with regard to the study design, the limitations of the study hinder an understanding of the occupational risks, including the failure to assess exposure to specific carcinogens. As multiple factors affected the likelihood of developing cancer, it was difficult to differentiate between occupation and lifestyle in this study. A statistically significant excess of cancer cases can occur within a given population without a discernible cause and might be a random occurrence. Further studies should explore the specific root cause in depth. Secondly, the healthy worker effect may have occurred due to the use of the general population as a comparison for study. The members of this group may change occupations or retire early because of cancer [30]. However, this study reviewed all of the cases and collected data from proxies on all deceased HCWs and 63% of them have survived and still work in the same healthcare setting for treatment. All of the cases were included in this analysis. In this way, the bias can be minimized. Thirdly, the population were observed over a 14-year period, but the expected number calculated over recent years has resulted in an increased incidence of cancer. At present, the results of the comparison could be an underestimation of the true number, although, the observed and expected number in this study still compared the same number of 100,000 person-years. Finally, the small number of cases such as leukemia and male physicians were based on three cases, which may also affect the results of the study. Some variables may also lack sufficient statistical power to identify risks. The stratification of each occupation by cancer type could not be connected to a specific exposure and may warrant further investigation.

HCWs are valuable workers who require a costly and high level of education. One problem for HCWs and their health protection were systematic control measures to minimize carcinogen exposure, hematologic carcinogens, such as risk assessment, which should be immediately included and implemented. They also need to address the issue of promoting safe work practices by their employing institutions [17]. To prevent cancer among HCWs, particularly physicians, the hospital should emphasize occupational health services and health promotion for HCWs to decrease the incidence of cancer rather than only offering routine services. Fortunately, nowadays the hospital accreditation system may help to improve and prevent such work-related health problems. Further studies may include identifying other risk factors of cancer in HCWs, such as individual health behavior, explaining the risks of a particular occupation or a specific type of cancer. These new epidemiological findings still require additional research to clarify the possible causes.

## Conclusion

This study lends some support to the hypothesis that the features of the occupations of HCWs may increase their risk of cancer. This study revealed that leukemia was higher than expected among HCWs and that physicians may have an increased risk of cancer than the general working population. However, interpretations should be made with caution due to the limited number of cases. Further studies should focus in detail on occupational exposures and behavior related to the risk of cancer. It may also be beneficial in terms of reducing the burden of work-related cancers and the well-being of HCWs.
